# Segment anything model (SAM)‐driven prompt‐guided interactive delineation of gross tumor volume for rectal cancer neoadjuvant radiotherapy

**DOI:** 10.1002/mp.70733

**Published:** 2026-10-21

**Authors:** Si Wu, Jianhao Geng, Lu Bai, Qi Chen, Shaobin Wang, Zhiyan Liu, Yi Du

**Affiliations:** ^1^ Institute of Medical Technology Peking University Health Science Center Beijing China; ^2^ Key Laboratory of Carcinogenesis and Translational Research (Ministry of Education/Beijing) Department of Radiation Oncology Peking University Cancer Hospital & Institute Beijing China; ^3^ MedMind Technology Co., Ltd Beijing China; ^4^ Department of Biomedical Engineering School of Medicine Tsinghua University Beijing China; ^5^ Inner Mongolia Engineering Center of Digital‐Intelligent Integrated Prevention and Screening of Malignant Tumors Proton Center & Department of Radiation Oncology Peking University Cancer Hospital (Inner Mongolia Campus)/Affiliated Cancer Hospital of Inner Mongolia Medical University Hohhot China

**Keywords:** deep learning, gross tumor volume, interactive segmentation, radiotherapy, rectal cancer, segment anything model (SAM)

## Abstract

**Background:**

Segment Anything Model (SAM), a prompt‐guided foundation model, exhibits substantial potential for medical image segmentation by incorporating prior knowledge through user‐defined prompts. However, the application of SAM in radiotherapy target delineation remains limited.

**Purpose:**

This study proposes a feasible prompt‐guided interactive delineation workflow driven by an optimized SAM.

**Methods:**

A total of 100 rectal cancer neoadjuvant radiotherapy cases were collected (60 for training and 40 for testing). This study utilized bounding‐box prompts to guide SAM for GTV segmentation. Physicians manually draw box prompts on certain slices, while box prompts for the remaining slices are automatically generated using an interpolation strategy. Four fine‐tuning strategies for SAM training were evaluated, and the impact of prompt numbers and placements was assessed as well. The performance of the finalized workflow with optimized SAM was compared with five state‐of‐the‐art (SOTA) models, and its robustness to prompt variations in middle‐slice selection and box‐prompt margins was analyzed.

**Results:**

Compared to other fine‐tuning methods, freezing the image encoder obtained the most stable performance. Prompts on only 3 slices achieved accuracy comparable to prompts on all slices. The placement of the middle‐slice prompt exhibited negligible impact (*p* > 0.05). The finalized SAM‐driven workflow outperformed end‐to‐end baseline models both visually and quantitatively (3D DSC: 0.92 vs 0.80; 3D HD95: 2.73 vs 12.70; IoU: 0.86 vs 0.67; Sensitivity: 91.20% vs 85.98%; Precision: 93.65% vs 79.23%), while maintaining stable performance across certain prompt variations.

**Conclusions:**

The proposed SAM‐driven interactive workflow with as few as 3 box prompts achieves favorable accuracy of GTV segmentation for rectal cancer neoadjuvant radiotherapy.

## 1 INTRODUCTION

Colorectal cancer ranks among the top three malignancies worldwide in terms of both incidence and mortality.^1^ Among the colorectal cancer cases in China, rectal cancer accounts for the largest proportion.^2^ For patients with locally advanced rectal cancer, neoadjuvant radiotherapy followed by surgery has become the recommended treatment regime,^3^ as it significantly reduces postoperative local recurrence and improves pathological complete response rates.^4^

As an integral component of radiotherapy, accurate delineation of target volumes (TVs) plays a crucial role in achieving favorable treatment outcomes.^5^ TVs delineation task of rectal cancer neoadjuvant radiotherapy includes two components: gross tumor volume (GTV) and clinical target volume (CTV). According to EORTC^6^ and NCCN guidelines,^7^ the GTV refers to the visible primary tumor and metastatic lymph nodes, and the CTV includes the GTV, subclinical lesion, and high‐risk lymphatic drainage areas. Traditional manual delineation of TVs on CT images is time‐consuming, labor‐intensive, and prone to human‐related errors.^8^, ^9^

To improve clinical efficiency, numerous efforts have been made towards automating TVs’ delineation. Over the past few years, deep learning (DL)‐based methods have emerged as the dominant approach. Several successful studies on automated CTV delineation in rectal cancer have been conducted. For example, Men et al.^10^ proposed a deep dilated CNN (DDCNN) model, which achieved a mean Dice Similarity Coefficient (DSC) value of 0.87. Song et al.^11^ utilized the DeepLabV3+^12^ network, of which the average volume and surface DSC achieved 0.88 and 0.79, respectively. Wu et al.^13^ developed a modified U‐Net model, achieving a mean DSC of 0.90. Our group^14^ also proposed a dual‐path network (DpnUnet) that reached a DSC of 0.85.

Compared with CTV, automated GTV delineation in rectal cancer remains particularly challenging due to the poor contrast of tumors in CT images, and there are limited works addressing this issue. Li et al.^15^ adopted nnU‐Net^16^ for all‐in‐one rectal cancer radiotherapy, with GTV DSC values ranging from 0.71 to 0.80. Geng et al.^17^ proposed a triple‐stage workflow by incorporating tumor location in MRI into CT images for GTV segmentation, achieving the highest mean DSC of 0.87 to date. Despite the top performance, the triple‐stage workflow is susceptible to MRI‐to‐CT registration errors, resulting in volume mismatches. This gap highlights the need for strategies that can robustly transfer cross‐modality information from MRI to CT, even in the presence of registration errors.

Recently, prompt‐guided models have emerged as a promising solution. By utilizing user‐defined prompts, these models facilitate the incorporation of patient‐specific a‐priori knowledge to improve segmentation performance. One of the most notable prompt‐guided models is Segment Anything Model (SAM),^18^ an open‐source large‐scale visual model proposed by Meta AI. Compared with conventional models, SAM is pretrained on an extensive dataset and allows users to annotate regions of interest (ROIs) via various interactions, such as points, bounding boxes, or mask prompts. Several studies^19^, ^20^, ^21^, ^22^ have applied SAM to medical image segmentation tasks, encompassing multiple imaging modalities such as CT, MRI, ultrasound, and X‐ray. These studies have demonstrated that SAM achieves satisfactory segmentation performance, particularly in organs or lesions with clear boundaries.

In radiotherapy, the application of SAM is quite limited and mostly in head and neck cancers for organ‐at‐risk (OAR) delineation.^23^, ^24^, ^25^, ^26^ To date, our literature search on target delineation yields only one study by Yang et al.^27^ performing MRI‐based pelvic tumor segmentation. While these studies indicate that SAM has significant potential for target delineation, they mainly focus on benchmarking model performance, and how to effectively integrate SAM into clinical target delineation workflows still remains unclear.

This is a pilot study that aims to propose a SAM‐driven prompt‐guided interactive workflow. By incorporating clinical prior knowledge through manually defined bounding box prompts, the workflow enhances CT‐based target delineation accuracy for rectal cancer. Particularly, in order to optimize the overall performance of the workflow, we systematically investigate the impact of several confounding issues, including fine‐tuning strategies, the optimal prompt number and placements, and robustness to prompt variability. As validation, the finalized SAM‐driven workflow was compared with five state‐of‐the‐art (SOTA) DL baseline models. The key novelty of this work is to propose a SAM‐driven prompt‐guided interactive workflow for GTV delineation on planning CT images for rectal cancer radiotherapy, which may be easily adapted into routine clinical practice.

The key contributions of this work include: (a) a feasible SAM‐driven interactive workflow for GTV delineation in planning CT, which requires limited user‐defined prompts; and (b) the optimal SAM fine‐tuning strategy, and recommended prompt number and placement, which lead to outperforming segmentation accuracy over baseline methods; and (c) validated robustness of prompt variations in middle‐slice selection and box‐prompt margin.

## 2 METHODS

### 2.1 Overview of segment anything model and proposed workflow

SAM is a transformer‐based 2D segmentation framework pretrained on the SA‐1B dataset, which contains over 11 million natural images and more than 1 billion segmentation masks. It includes three main components: an image encoder, a prompt encoder, and a mask decoder. The image encoder is a pretrained Vision Transformer (ViT) backbone available in three scales (ViT‐B, ViT‐L, and ViT‐H). Owing to its large‐scale pretraining, the encoder exhibits strong generalization capabilities across diverse segmentation tasks. The prompt encoder converts user‐defined prompts into feature embeddings that can be aligned with visual representations, enabling SAM to flexibly incorporate different forms of guidance. The mask decoder is a lightweight transformer module that integrates features from both encoders via a cross‐attention mechanism. This structure enhances representation learning by effectively capturing long‐range dependencies, which contributes to improving accuracy compared with conventional CNN‐based decoders. SAM supports three types of prompts for user interaction: points, boxes, and masks. Point prompts include both foreground points and background points, indicating regions to be included or excluded in the segmentation. Box prompts define a rectangular region of interest that guides the model to focus on the enclosed area. Mask prompts serve a similar function as boxes by providing prior information about the target shape.

In this study, we adopted a pretrained SAM with a ViT‐B backbone^18^ as the base model. The overall SAM‐driven prompt‐guided interactive workflow of GTV delineation is depicted in Figure 1 as well as the general study design. The workflow for clinical practice is composed of four steps:

- **Step 0** (preparation): physicians learn the general tumor information from medical records (for instance, diagnostic images or reports) as a‐priori knowledge.
- **Step 1**: physicians identify the general target volume in planning CT and annotate the bounding boxes of GTV at least on the superior and inferior slices.
- **Step 2**: box prompts that are not manually annotated on the intermediate slices are automatically generated by inter‐slice interpolation.
- **Step 3**: annotated CT images as well as the associated box prompts are sequentially input to SAM to generate desired GTV contours.

**FIGURE 1:**
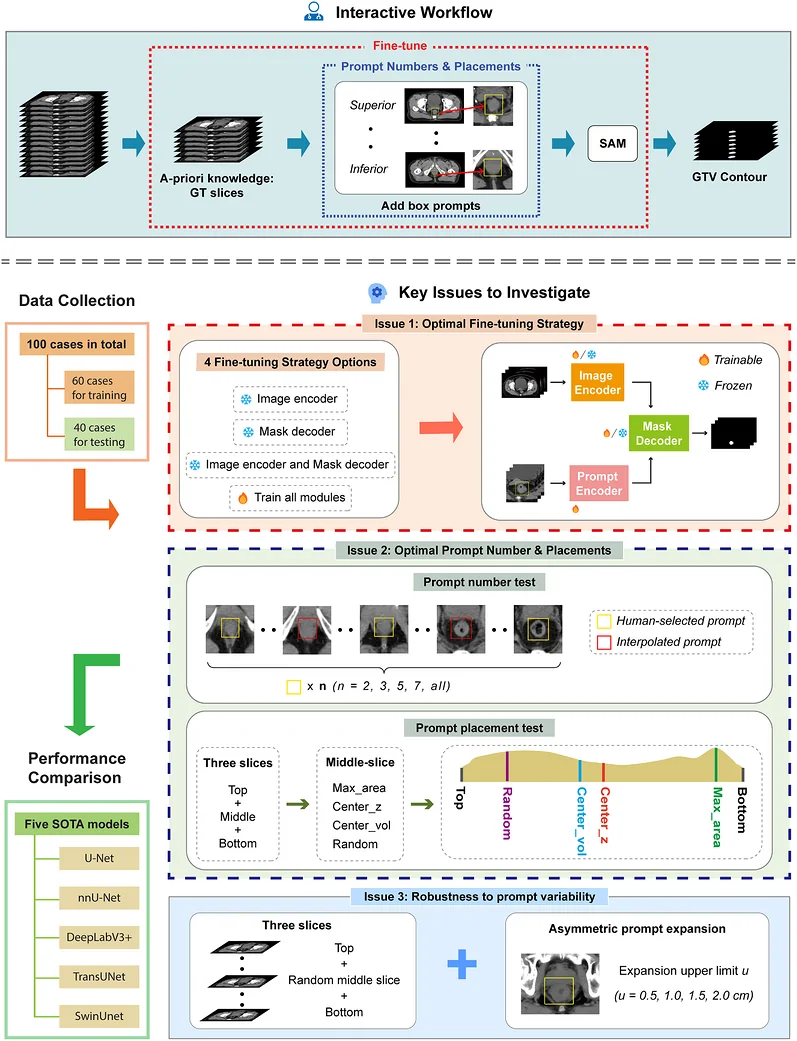
Overview of the proposed SAM‐driven prompt‐guided workflow and study framework. The lower panels summarize the study framework, including data collection, three investigated key issues, and a performance comparison experiment. In Issue 1, four fine‐tuning strategies are used in training. Issue 2 consists of two parts, varying the number and placement of interactive prompts under an interpolation scheme. In the rightmost subfigure of the prompt placement test panel, the horizontal axis represents the placement from superior to inferior slice, and the curve height indicates the tumor area. Issue 3 shows the prompt variability analysis, including a randomly selected middle slice and asymmetric in‐plane prompt expansions.

It is important to note that herein we used a pretrained SAM as the base model, and adapted the model capability specifically by localized retraining. As shown in Figure 1, three key issues need to be addressed to finalize the workflow: (1) optimal fine‐tuning strategy for SAM training; and (2) required prompt numbers and placements in SAM‐driven inference; and (3) the robustness to prompt variability.

### 2.2 Data acquisition and preprocessing

This retrospective study was approved by the institutional review board of Peking University Cancer Hospital. A total of 100 patients with Stage II to III mid‐low rectal cancer without lymph node metastasis were collected. All patients underwent neoadjuvant radiotherapy between 2020 and 2025. The planning CT images and contouring structures were collected in a fully anonymized approach without any health records. The patient cohort was divided into a training set (60/100) and a testing set (40/100) by random sampling (Fisher–Yates shuffle). The median number of planning CT slices per patient was 88 (range: 72–122) in the training cohort and 97.5 (range: 81–119) in the testing cohort. Among these slices, the median number of slices with GTV contours was 10 per patient (range: 4–17) in the training cohort and 10 per patient (range: 7–15) in the testing cohort.

All the patients underwent CT simulations in the supine posture. The CT images were acquired on two big‐bore RT‐specific CT scanners (Siemens SOMATOM Sensation Open and Philips Brilliance Big‐bore). Among the entire cohort, 87 cases were acquired on the Siemens scanner and 13 on the Philips scanner. The training/testing cohorts included 50/37 cases scanned on Siemens and 10/3 cases on Philips respectively by random sampling. All the cases were scanned with a tube voltage of 120 kVp, a slice thickness of 5 mm, and an axial matrix size of 512 × 512. For the Siemens scanner, the spatial resolution was 1.27 mm × 1.27 mm with a field of view (FOV) of 65 cm, and images were reconstructed using a B20s convolution kernel. For the Philips scanner, the spatial resolution was 1.17 mm × 1.17 mm with a FOV of 60 cm, and images were iteratively reconstructed using iDose4 (Level = 4). For each case, tube current modulation (Siemens CareDose or Philips Dose Right) was applied.

CT images were first exported from the treatment planning system (Eclipse 16.1) in DICOM (.dcm) and then preprocessed into NIfTI (.nii) files for SAM training and testing. Specifically, for each slice, the gray‐scale was first normalized by a window width and window level of [350 HU, 40 HU], and then resized and duplicated as a three‐channel tensor of 3 × 1024 × 1024.

### 2.3 Fine‐tuning strategy for SAM training

To investigate the adaptability and performance of SAM for the GTV delineation task, we adopted multiple training configurations, as shown in Figure 1: (1) freezing the image encoder, (2) freezing the mask decoder, (3) simultaneously freezing the image encoder and mask decoder, (4) training all parameters. For the frozen modules, the pretrained parameters were kept fixed during training, while the other modules remained trainable. Model training was conducted using five‐fold cross‐validation, and the checkpoint with the best validation performance was selected for subsequent evaluation on the testing cohort.

To accommodate clinically feasible box prompts with relaxed boundary constraints, we adopted an in‐plane expansion strategy based on the GT bounding boxes. Specifically, during the training process, the four sides of box prompts were independently and randomly expanded in‐plane by 0–1 cm. As for the cross‐validation data, a fixed expansion of 0.5 cm of four sides was applied to ensure stable model selection. During the testing process, a series of testing cases with different fixed prompt expansions on all four sides were generated, ranging from 0 to 1.0 cm in 0.1 cm increments, with additional evaluations at 1.2 and 1.5 cm. The fine‐tuning strategy exhibiting stable and reasonable accuracy across expansion values was preferred for desired robustness. The preferred fine‐tuning strategy with the best checkpoint was selected as the optimal SAM configuration.

Based on the optimal configuration, we performed two supplementary analyses to further investigate SAM fine‐tuning under limited data conditions. First, we assessed the impact of training dataset size by randomly sampling 10, 20, 30, 40, 50, and 60 cases from the original training cohort. Second, low‐rank adaptation (LoRA)^28^ was introduced as an additional fine‐tuning strategy and compared with the four training configurations. For both experiments, the training and testing protocols were kept consistent with the fine‐tuning experiment described above. Detailed experimental setups and results are provided in the Supplementary Materials (Figure S 1, Figure S 2, and Table S 1).

The SAM training used AdamW optimizer and ReduceLROnPlateau scheduler. The initial learning rate was set to 0.001 and was reduced by a factor of 0.5 if the validation loss did not improve for 7 consecutive epochs. The weight decay was set to 0.0001, and the batch size was 2. The loss function was a combination of binary cross‐entropy and Dice loss (BCE–Dice loss) defined as:

(1) $$\mathrm{Loss}=0.5L_{\mathrm{BCE}}+0.5L_{\mathrm{Dice}}$$

(2) $$\;L_{\mathrm{BCE}}=-\frac{1}{N}\sum_{i\;=1}^{N}[y_{i}\log({\hat{y}}_{i})+(1-y_{i})\log(1-{\hat{y}}_{i})]$$

(3) $$\;L_{\mathrm{Dice}}=1-\frac{2\sum_{i\;=1}^{N}y_{i}{\hat{y}}_{i}}{\sum_{i\;=1}^{N}y_{i}+\sum_{i\;=1}^{N}{\hat{y}}_{i}}$$

where *L*_BCE_ and *L*_Dice_ denote the binary cross‐entropy loss and Dice loss, respectively. $y_{i}$ and ${\hat{y}}_{i}$ represent the ground truth mask and the predicted probability for the i‐th pixel, respectively. *N* is the total number of pixels.

An early stopping strategy was employed to prevent overfitting, and the maximum epoch was 100. Gradient clipping with a threshold of 0.1 was applied to stabilize training. All the networks were implemented in PyTorch 2.6.0 with CUDA 11.8 and trained on an NVIDIA A100 GPU.

### 2.4 Prompt numbers and placements in SAM‐driven inference

In model inference, sparser manually annotated box prompts indicate less physician interaction, leading to increased clinical efficiency. To reduce interaction burden while maintaining reliable segmentation performance, we performed two experiments on the testing cohort by varying the numbers or the spatial placements of manual prompts.

In the number study, five configurations of manually prompted slice numbers (2, 3, 5, 7, and all slices) were set. The number of manual prompt slices corresponds to the frequency of physician interactions. Manual prompts were provided according to the predefined prompt numbers. The superior and inferior slices of the GTV served as mandatory prompt locations, and additional manual prompt slices were uniformly distributed within the GTV extent. Box prompts were expanded in‐plane by 0.5 cm on all four sides based on the GT bounding boxes on these manually selected prompt slices. Due to the relatively continuous morphology and limited inter‐slice variation commonly observed in rectal cancer GTV,^29^ an interpolation strategy was adopted for the slices without manual prompts. Specifically, box prompts were automatically generated through slice‐wise linear interpolation between the nearest manual prompt slices.

Based on the prompt number experiment, we further investigated the optimal placement of the middle‐slice prompt under the three‐box prompting strategy (the superior slice, the inferior slice, and the middle slice). In this experiment, one middle prompt slice was selected between the superior and inferior GTV slices. Since manual selection of the middle‐slice prompt may introduce inter‐observer variability in slice positioning, we designed four placement candidates for the middle‐slice prompt as shown in Figure 1: the central slice of the GTV along the z‐axis (center_z), the slice with the largest GTV area (max_area), the volumetric center slice of the GTV (center_vol), and a randomly selected slice within its extent (random). To simulate variability in clinical prompt inputs, the random slice selection was repeated ten times for all cases in the testing cohort using uniform random sampling with a fixed random seed.

### 2.5 Comparison of finalized workflow with baseline models

We used the well fine‐tuned SAM model in Section 2.3 and the recommended prompt number and prompt placement in Section 2.4 in the finalized workflow. Box prompts were generated using the expansion and interpolation strategies described in Section 2.4. To validate the effectiveness of the proposed SAM‐driven workflow, we selected five SOTA models as the baseline for comparison. The SOTA models are U‐Net,^30^ DeepLabV3+,^12^ nnU‐Net,^16^ TransUNet,^31^ and SwinUnet.^32^ Among these SOTA models, the first three demonstrate prevailing performance in automated CTV delineation for rectal cancer radiotherapy, and the last two are popular and top‐performing models in several recent segmentation studies.^33^, ^34^, ^35^ All baseline models were trained and evaluated on the same dataset as the SAM model. We adopted five‐fold cross‐validation, 100 training epochs, and specific hyperparameter settings for each model. It is important to note that all baseline models are fully automatic segmentation approaches without user interaction. The primary objective of this comparison is to evaluate the effectiveness of prompt‐guided interactive workflow against conventional automatic segmentation paradigms.

### 2.6 Robustness to prompt variability

As described in Section 2.1, physicians are required to manually place box prompts on certain slices, during which prompt variability inevitably exists in the coverage margins around the GT region. To evaluate the robustness of the proposed workflow to prompt variability, we conducted a prompt variability sensitivity analysis. In this experiment, we adopted the same fine‐tuned SAM model, prompt number, prompt placement, and interpolation strategy as in Section 2.5. The procedure was as follows:

1. For each patient in the testing cohort, the middle slice was randomly selected within the GTV extent using discrete uniform sampling, and this operation was repeated ten times.
2. For each middle‐slice selection, four asymmetric box prompt expansion settings were evaluated. Specifically, the four sides of box prompts were independently and randomly expanded in‐plane based on the GT bounding boxes. The expansion distance *d* for each side was sampled from a uniform distribution *d* ∼ U(0,*u*), where *u* denotes the expansion upper limit and was set to 0.5, 1.0, 1.5, and 2.0 cm, respectively. Accordingly, the expected value of the expansion distance was *E(d)* = *u*/2, corresponding to 0.25, 0.5, 0.75, and 1.0 cm, respectively.
3. Box prompts were generated using the interpolation strategy for non‐prompted slices.
4. For each expansion distance setting, the performance was averaged over ten random middle‐slice selections across all testing patients.

### 2.7 Evaluation metrics

The evaluation metrics we used herein are the Dice Similarity Coefficient (DSC), 95th percentile Hausdorff Distance (HD95), Intersection over Union (IoU), Average Surface Distance (ASD), Sensitivity, and Precision.^36^ Specifically, DSC and IoU are used to assess the overlap between the predicted and GT masks. HD95 and ASD measure the boundary accuracy of the segmentation. Sensitivity reflects the extent of missed tumor voxels, and precision reflects the extent of over‐segmented voxels. Note that DSC and HD95 values were computed at both slice‐wise (2D) and volumetric (3D) levels.

### 2.8 Statistical analysis

All quantitative results were reported as mean ± standard deviation. Non‐parametric tests were applied to DSC, HD95, IoU, and ASD in this study. In the testing study (Section 2.4) to evaluate the impact of prompt numbers and middle‐prompt placements, the Friedman test was applied to examine overall differences across prompt numbers and placements. If the Friedman test indicated that the difference is statistically significant (*p* < 0.05), the post‐hoc Nemenyi tests were then conducted to evaluate pairwise differences between the prompt settings. In the comparison study (Section 2.5), we used the Wilcoxon signed‐rank test to compare the fine‐tuned SAM model with the five SOTA models. We performed all data analysis using OriginPro 2024 (OriginLab Corporation, USA).

## 3 RESULTS

### 3.1 Optimal fine‐tuning strategy

The performance of the four fine‐tuning methods (freezing the image encoder, freezing the mask decoder, freezing both the image encoder and mask decoder, and training all modules) was evaluated across different bounding box expansion values (Figure 2). For the DSC metric (Figure 2a,b), higher values indicate more overlap between the predicted contours and the GT, whereas for the HD95 metric (Figure 2c,d), lower values indicate smaller boundary deviations between the predicted contours and the GT.

**FIGURE 2:**
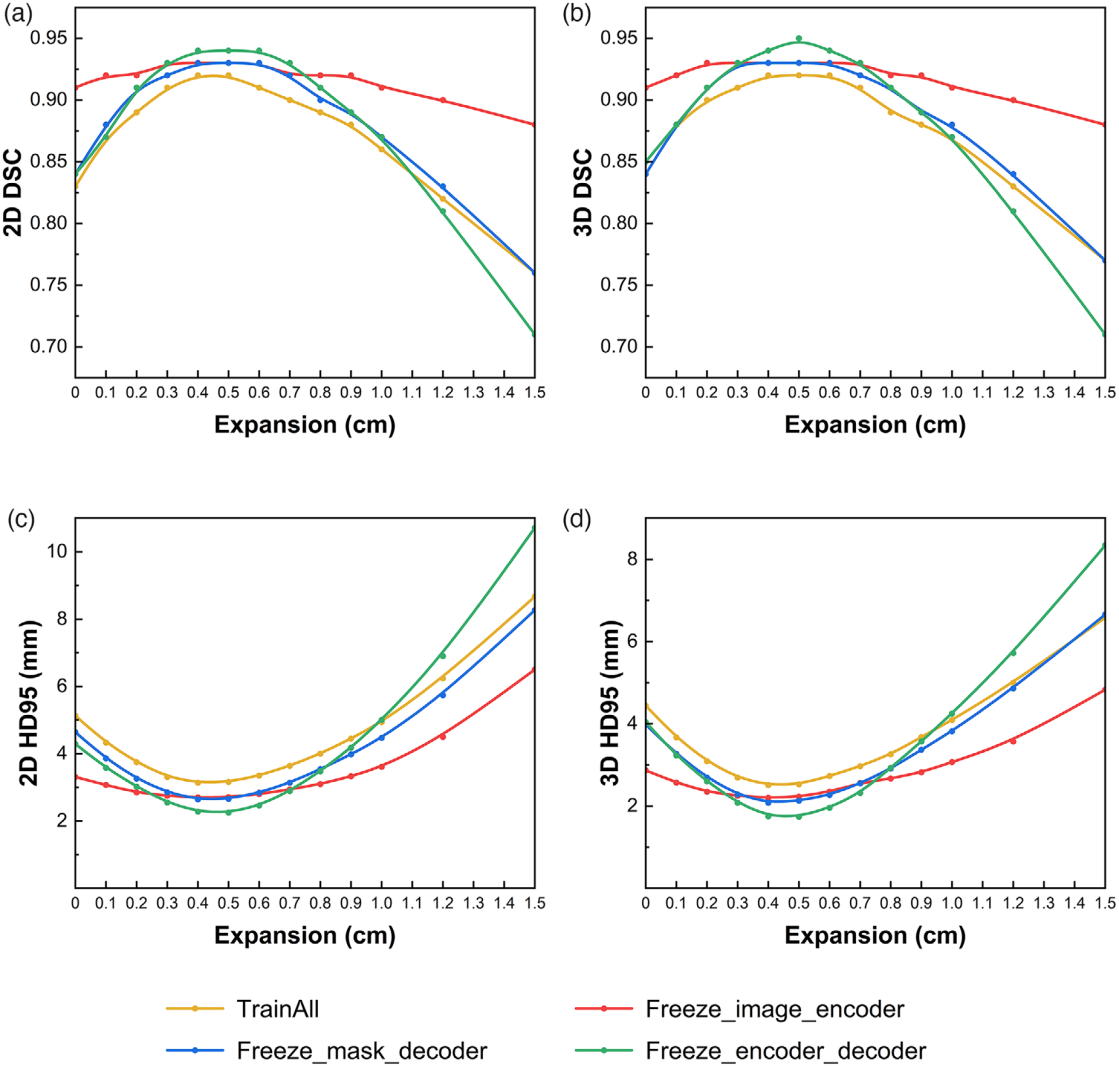
Comparison of DSC (a–b) and HD95 (c–d) metrics across different bounding box expansion values for four fine‐tuning strategies of the SAM‐ViT‐B model. TrainAll: training all parameters; Freeze_image_encoder: the image encoder was frozen; Freeze_mask_decoder: the mask decoder was frozen; Freeze_encoder_decoder: both the image encoder and mask decoder were frozen.

As shown in Figure 2, the four fine‐tuning strategies exhibited similar trends: with the box expansion, DSC values first increased and then decreased, while HD95 first decreased and then increased. The detailed performance ranges for fine‐tuning strategies are summarized in Table 1. Among the four strategies, freezing the image encoder achieved the smallest value fluctuations while maintaining relatively high performance in both DSC and HD95 across different expansion distances (2D DSC: 0.88–0.93, 3D DSC: 0.88–0.93, 2D HD95: 2.70–6.50, 3D HD95: 2.20–4.83), particularly within the 0–1 cm interval. Specifically, for the strategy of freezing the image encoder, the best results were achieved with an expansion range of 0.3–0.6 cm, and all metrics exhibited a substantial decline when the expansion exceeded 1 cm.

**TABLE 1:** Performance ranges of four SAM fine‐tuning strategies under varying box expansion margins (0–1.5 cm).

Fine‐tuning Strategy	2D DSC	3D DSC	2D HD95 (mm)	3D HD95 (mm)
TrainAll	0.76–0.92	0.77–0.92	3.14–8.67	2.51–6.58
Freeze_image_encoder	0.88–0.93	0.88–0.93	2.70–6.50	2.20–4.83
Freeze_mask_decoder	0.76–0.93	0.77–0.93	2.64–8.27	2.08–6.66
Freeze_encoder_decoder	0.71–0.94	0.71–0.95	2.25–10.71	1.74–8.34

* Note: Evaluation values are presented as ranges (minimum–maximum). TrainAll: training all parameters; Freeze_image_encoder: the image encoder was frozen; Freeze_mask_decoder: the mask decoder was frozen; Freeze_encoder_decoder: both the image encoder and mask decoder were frozen.

### 3.2 Impact of prompt numbers

Figure 3 shows the evaluation metrics of providing different prompt numbers in testing. Using only 2 slices showed the poorest performance across metrics (3D DSC: 0.91 ± 0.04; 3D HD95: 3.42 ± 1.83; IoU: 0.83 ± 0.06; ASD: 0.95 ± 0.36). 3_slices (3D DSC: 0.93 ± 0.02; 3D HD95: 2.54 ± 1.04; IoU: 0.87 ± 0.04; ASD: 0.82 ± 0.28) markedly improved performance and showed comparable results to all_slices (3D DSC: 0.93 ± 0.02; 3D HD95: 2.23 ± 0.90; IoU: 0.87 ± 0.03; ASD: 0.81 ± 0.28). Statistical analysis showed a significant overall difference across prompt numbers, as shown by the Friedman test in Figure 3. The post‐hoc Nemenyi test indicated that no significant differences were found between 3_slices, 5_slices, 7_slices, and all_slices in metrics except HD95. As shown in Figure 3c,d, 3_slices showed differences from 7_slices and all_slices in HD95, but the values and overall distribution shapes were highly similar in these three settings, indicating that these differences are unlikely to be clinically meaningful.

**FIGURE 3:**
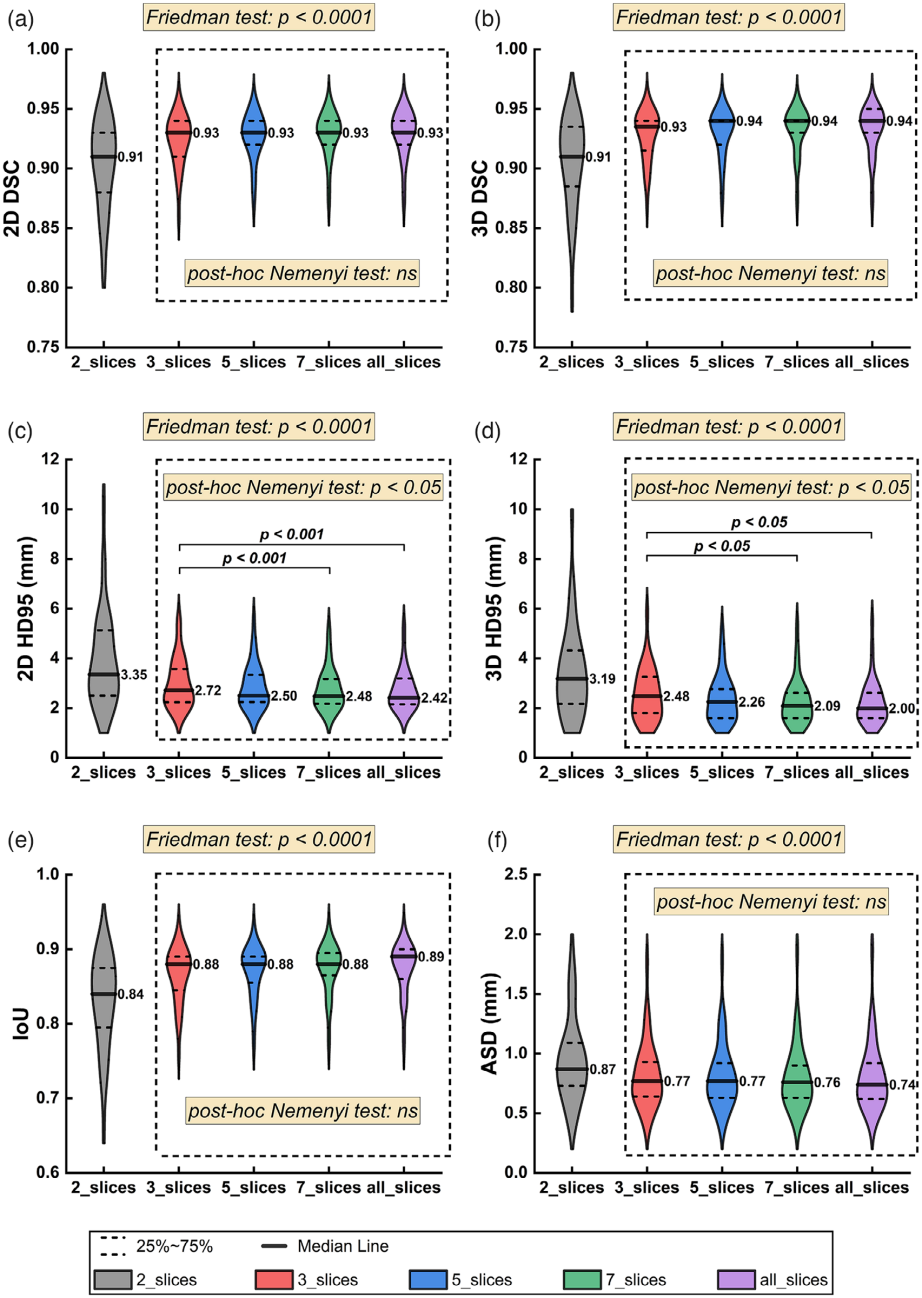
Comparison of evaluation metrics across different prompt numbers for fine‐tuned SAM. The numbers within each violin plot represent the median values of each group. Statistical significance was evaluated using the Friedman test with post‐hoc Nemenyi correction; *ns: not significant*.

### 3.3 Impact of middle‐slice prompt placement

Table 2 shows the performance of different middle‐slice placement options based on the three‐box prompting strategy. The four options (center_z, max_area, center_vol, and random) showed similar results in all evaluation metrics, and no statistically significant differences were observed (*p* > 0.05).

**TABLE 2:** Evaluation results (mean ± SD) of fine‐tuned SAM with three‐box prompting under different middle‐slice selection strategies on the testing cohort.

	DSC		HD95 (mm)					
Prompt Placement	2D	3D	2D	3D	IoU	ASD (mm)	Sensitivity (%)	Precision (%)
center_z	**0.92 **± 0.02	**0.93 **± 0.02	**2.96 **± 0.94	**2.53 **± 1.00	**0.87 **± 0.04	**0.82 **± 0.27	**92.26 **± 3.94	93.59 ± 3.55
max_area	**0.92 **± 0.02	**0.93 **± 0.02	3.06 ± 0.95	2.61 ± 0.97	0.86 ± 0.04	0.84 ± 0.27	92.11 ± 4.25	93.31 ± 3.70
center_vol	**0.92 **± 0.02	**0.93 **± 0.02	2.98 ± 0.95	2.59 ± 1.04	**0.87 **± 0.04	**0.82 **± 0.27	92.17 ± 4.10	93.53 ± 3.64
random	**0.92 **± 0.02	0.92 ± 0.02	3.20 ± 1.15	2.73 ± 1.12	0.86 ± 0.04	0.86 ± 0.29	91.20 ± 4.52	**93.65 **± 3.56
*p*‐value	> 0.05	> 0.05	> 0.05	> 0.05	> 0.05	> 0.05	> 0.05	> 0.05

* Note: Bold values indicate the best mean value among the four middle‐slice prompt placement strategies. The Friedman test indicates no statistically significant differences among four middle‐slice locations (*p* > 0.05).

### 3.4 Comparison with SOTA models

As shown in Figure 4, the proposed SAM performance was averaged over ten random middle‐slice selections for all testing patients. The results were compared with representative DL segmentation models. Our proposed SAM workflow achieved the best performance across all evaluation metrics. Compared with the best baseline values (2D DSC: 0.84; 3D DSC: 0.80; 2D HD95: 3.60; 3D HD95: 12.70; IoU: 0.67; ASD: 3.62; Sensitivity: 85.98%; Precision: 79.23%), the proposed SAM improved the 2D DSC, 3D DSC, 2D HD95, 3D HD95, IoU, ASD, Sensitivity, and Precision to 0.92, 0.92, 3.20, 2.73, 0.86, 0.86, 91.20%, and 93.65%. No significant difference was observed for 2D HD95 between the proposed SAM and the nnU‐Net and U‐Net models (*p* > 0.05). For all other evaluation metrics, SAM achieved statistically superior performance.

**FIGURE 4:**
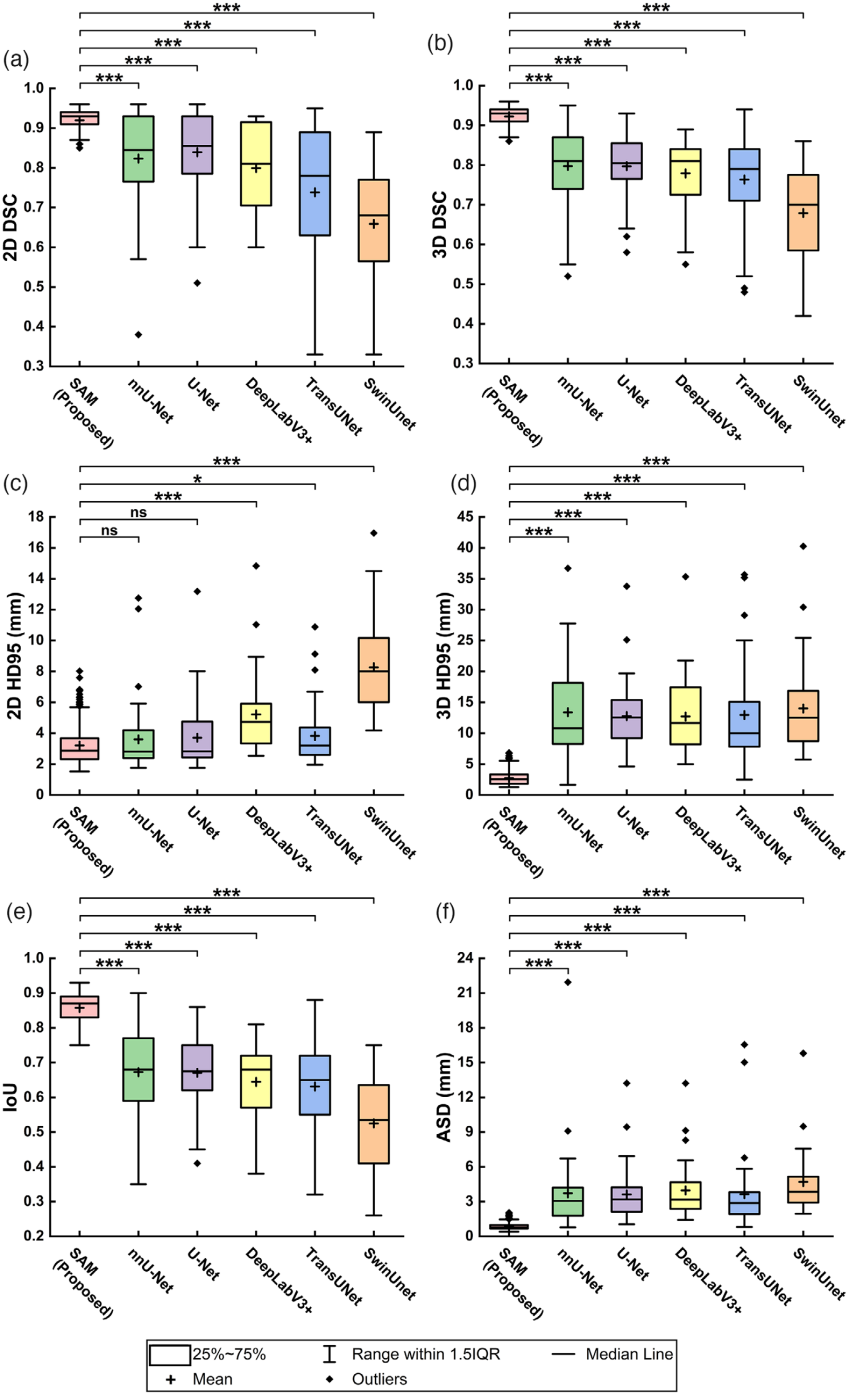
Comparison of our proposed SAM workflow with five SOTA models. The proposed SAM represented the averaged results of ten random middle‐slice selections for 40 patients in the testing cohort. Statistical significance was calculated using the Wilcoxon signed‐rank test: * p ≤ 0.05; ** p ≤ 0.01; *** p ≤ 0.001; ns: not significant.

Figure 5 presents visual comparisons of the GTV contours delivered by different models in four representative cases (Rows 1–4). The same case image was displayed for all models in each row. For the proposed SAM, a representative prediction was selected from the ten random middle‐slice tests for each case. Specifically, the prediction closest to the mean evaluation values reported for the random setting in Table 2 was used for visualization. We can clearly see that the contours produced by SAM demonstrated excellent alignment with the GT, while the comparison models presented evident discrepancies. In Rows 1 and 2 where the target boundaries were indistinct, most baseline models exhibited over‐segmentation results. In Row 3 where the target area was irregular, baseline models failed to capture the accurate shape, especially for nnU‐Net. In Row 4 where the target area was larger than in typical cases, under‐segmentation errors were observed in the baseline models.

**FIGURE 5:**
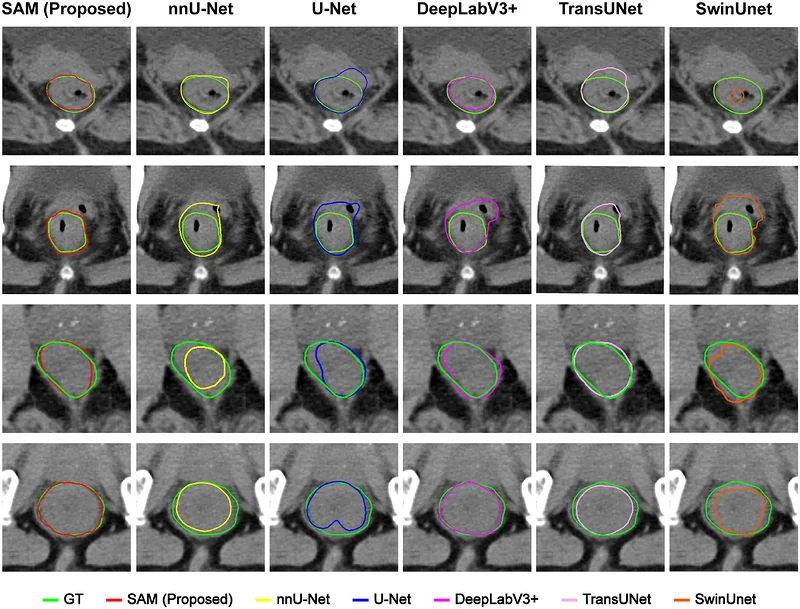
Visual comparison of GTV segmentation results contoured by SAM (Proposed) and baseline models for four representative cases. Each row corresponds to one case, and different columns represent different models. The GT (green contour) represents the ground truth contour used as the reference standard.

### 3.5 Prompt variability analysis

The results of the prompt variability experiment are summarized in Table 3. As the random expansion limit became larger, sensitivity values increased while precision decreased, indicating a gradual shift from under‐contouring to over‐contouring. As for the other metrics, the proposed workflow achieved the best overall performance under the Random 0–1.0 cm setting, with the highest DSC and IoU values (2D DSC: 0.92; 3D DSC: 0.92; IoU: 0.85), and the lowest HD95 values (2D HD95: 3.39; 3D HD95: 2.90). The metrics showed minor variation under the Random 0–0.5 cm and the Random 0–1.5 cm settings. The Random 0–2.0 cm setting showed the worst HD95 and ASD values (2D HD95: 4.90; 3D HD95: 4.22; ASD: 1.59).

**TABLE 3:** Evaluation results (mean ± SD) of the proposed SAM workflow with three‐box prompting interpolation under different prompt expansion settings on the testing cohort.

	DSC		HD95 (mm)					
Expansion Setting	2D	3D	2D	3D	IoU	ASD (mm)	Sensitivity (%)	Precision (%)
Random 0–0.5	0.91 ± 0.03	0.91 ± 0.03	3.59 ± 1.38	3.19 ± 1.32	0.84 ± 0.05	**0.88 **± 0.30	87.63 ± 5.54	**95.38 **± 2.72
Random 0–1.0	**0.92 **± 0.02	**0.92 **± 0.02	**3.39 **± 1.16	**2.90 **± 1.12	**0.85 **± 0.04	0.91 ± 0.30	90.78 ± 4.45	93.34 ± 3.53
Random 0–1.5	0.91 ± 0.03	**0.92 **± 0.03	3.68 ± 1.22	3.16 ± 1.20	**0.85 **± 0.04	1.08 ± 0.40	92.54 ± 3.91	90.04 ± 4.64
Random 0–2.0	0.90 ± 0.03	0.90 ± 0.03	4.90 ± 1.60	4.22 ± 1.64	0.83 ± 0.05	1.59 ± 0.65	**93.54 **± 3.61	87.87 ± 6.02

* Note: Random 0–u (u = 0.5, 1.0, 1.5, 2.0 cm): the four sides of the box prompts were independently and randomly expanded in‐plane from 0 to u cm based on the GT bounding boxes. Bold values indicate the best mean value among the four prompt expansion settings averaged over ten random middle‐slice selections.

## 4 DISCUSSION

Deep‐learning‐based GTV delineation for rectal cancer remains challenging due to the poor soft tissue contrast of CT images. To address this issue, this study proposes a prompt‐guided interactive workflow using a fine‐tuned SAM model. The workflow allows physicians to guide GTV delineation by providing sparse box prompts, achieving superior accuracy (DSC: 0.92) over current DL models and previous GTV delineation studies^15^, ^17^ (DSC: 0.71–0.87). However, the use of SAM as the backbone induced several confounding factors, and their impact on overall performance should be evaluated to further optimize the workflow.

In this study, we first investigated the fine‐tuning strategy for pretrained SAM. Among the four fine‐tuning strategies, freezing the image encoder showed the most stable performance across different box expansion values, which indicates strong tolerance to box prompt margins. Also, expanding the box margin beyond 1 cm led to a gradual decline in segmentation performance, suggesting that overly loose box prompts may induce unnecessary background information and consequently corrupt the segmentation accuracy.

Meanwhile, it is important to note that the strategy of training all modules exhibited inferior performance. While this finding seems counter‐intuitive, it is consistent with a previous study^22^ using SAM on 2D endoscopic polyp segmentation (PP‐SAM), which suggested that full‐parameter fine‐tuning may not be preferable for SAM adaptation with a limited dataset, and that freezing certain pretrained modules can help reduce the risk of overfitting and improve model performance. This may be attributed to the limited dataset size. Specifically, since the SAM model was originally trained on an extensive dataset, fully fine‐tuning on a much smaller dataset probably disrupted the pretrained general visual representations and therefore potentially led to catastrophic forgetting.^37^ To verify this, we performed additional experiments by introducing LoRA modules. By inserting trainable low‐rank matrices into the image encoder, LoRA‐based fine‐tuning of SAM outperformed the TrainAll strategy and achieved comparable performance to the Freeze_image_encoder strategy within the 0–1 cm box expansion range (Table S1, Figure S2). However, the fine‐tuning strategy recommended by PP‐SAM was not consistent with ours. PP‐SAM found that freezing the mask decoder achieved both the optimal and the most stable performance. This inconsistency may be attributed to differences in imaging modality and target complexity. These findings further indicate that the choice of fine‐tuning strategy significantly affects the model's adaptability to different tasks and prompt expansions, and thus should be tailored to specific task characteristics. Overall, for the task of rectal cancer GTV segmentation herein, freezing the image encoder while training the prompt encoder and mask decoder exhibits an appropriate balance between preserving pretrained visual representations and enabling task‐specific adaptation.

As one of the key novelties, the proposed workflow requires manual box prompts on as few as three slices (the superior slice, the inferior slice, and one intermediate slice) to perform GTV segmentation with near‐optimal performance. Since previous SAM‐driven studies^26^, ^38^ in target segmentation focused on evaluating the upper limit performance of SAM under dense slice prompts, to the best of our knowledge, this is the first study that proposed a feasible workflow with sparse prompts to achieve superior performance. This may be attributed to the underlying prompt interpolation scheme. Moreover, the results in Section 3.2 show that providing prompts on all slices can obtain the best performance. The minimal requirement of user interactions and the freedom of adding prompts may bring potential benefits to clinical efficiency. For example, in scenarios that are sensitive to time cost, such as online adaptive radiotherapy, physicians only need to perform three prompts, significantly reducing the interaction burden. For clinically complex cases with challenging tumor morphology, such as indistinct boundaries, highly irregular contours, or pronounced inter‐slice variation, additional prompts can be added to improve delineation accuracy.

As for the three‐prompt requirement, we found that the placement of the middle‐slice prompt presents negligible impact. It suggests that physicians can obtain desirable contouring results without having to precisely identify the specific intermediate slice. The effectiveness of our three‐slice prompt interpolation strategy may be attributed to two aspects. First, the rectal GTV is characterized by a continuous mass extending along the rectal wall on CT images.^6^, ^7^ Due to this growth pattern, the GTV morphology exhibits gradual changes across adjacent slices.^29^ As a consequence, variations in middle‐slice selection may generate minor differences under the interpolated prompt design. Second, the box prompt in SAM serves as a spatial cue rather than a strict boundary constraint. During inference, box prompts interact with image embeddings through a cross‐attention mechanism, guiding the model to focus on relevant regions of the image. Importantly, the final prediction of the mask decoder is still based on the entire image feature information. This design enables the model to infer beyond the prompt region, integrating both the prompt and the global image information to achieve accurate segmentation.

To validate the proposed workflow, we compared it with five end‐to‐end baseline models. Compared with these fully automated segmentation methods, the SAM‐driven workflow is a semi‐automatic approach that incorporates prompt inputs. Prior to this study, Li et al.^15^ used nnU‐Net to delineate GTV with DSC values ranging from 0.71 to 0.80. Geng et al.^17^ proposed a triple‐stage workflow based on MRI‐to‐CT spatial mapping. While the triple‐stage workflow achieves a DSC of 0.87, it is prone to residual errors in MRI‐to‐CT registration. Compared with these counterpart studies, this work is free from MRI‐to‐CT registration errors and achieves the highest mean DSC of 0.92. This prompt‐guided paradigm may reduce the burden of slice‐by‐slice manual delineation by improving segmentation accuracy. Particularly, in some complex cases as shown in Figure 5, integration of specific prompt guidance brings significant benefits in generalized efficacy.

To evaluate the workflow robustness to prompt variability, we designed a randomized test to simulate variations in middle‐slice selection and the associated box prompts. The group of Random 0–1.0 demonstrated balanced performance over the metrics. Note that the four sides of box prompts in Random 0–1.0 cm were expanded in‐plane by uniformly sampling within 0–1.0 cm, which means the expected value of side expansion was 0.5 cm. This finding is consistent with the box expansion margin test (Table 2). This further indicates that the workflow is robust to prompt variability, which may facilitate clinical application without the need for highly standardized prompts.

As a pilot study, the present work has several notable limitations. First, this is a single‐center study with a limited sample size. To better assess the generalizability of our proposed workflow, future studies will enlarge the study cohort and perform external validation. Second, due to the limitations of retrospective data acquisition, CT images in this study were obtained from two different scanners. Differences in scanner vendors and acquisition protocols may affect image textures and model performance. More standardized image acquisition protocols may help reduce such variability. Third, the baseline SAM is a 2D segmentation model. Although we adopted an inter‐slice interpolation strategy and achieved favorable volumetric evaluation results, there may still be issues with insufficient inter‐slice continuity and poor contour smoothness in some cases. In addition, newer variants have recently emerged, such as SAM2,^39^ and MedSAM.^40^ Future work could investigate newer variants to explore 3D interactive segmentation frameworks. Finally, although prompt variability was simulated in this study, whether the expansion distance for each side of the prompt aligns with inter‐observer variations should be further evaluated. Additionally, due to the lack of an off‐the‐shelf interactive interface, all testing was conducted at the code level. Future work will develop an interactive interface to evaluate multi‐physician variability and actual interaction efficiency.

## 5 CONCLUSIONS

The proposed SAM‐driven prompt‐guided interactive workflow enables accurate and robust GTV delineation for rectal cancer using minimal physician interaction. With only three bounding‐box prompts, the workflow achieves superior accuracy compared with conventional DL models, demonstrating the potential and feasibility for practical application in radiotherapy target delineation.

## CONFLICT OF INTEREST STATEMENT

The authors have no relevant conflicts of interest to disclose.

## Supporting information



Supporting information

## Data Availability

The data that support the findings of this study are available from the corresponding author upon reasonable request.
