# Supplementary material for: Segment anything model (SAM)‐driven prompt‐guided interactive delineation of gross tumor volume for rectal cancer neoadjuvant radiotherapy
Source: Med Phys. 2026 Oct 21;53(11):e70733. doi: 10.1002/mp.70733 (PMC13686343; doi:10.1002/mp.70733)
Supplement: Supplementary file 1 [file MP-53-0-s001.docx]

The impact of training dataset size is shown in Figure S1.

**Figure S1** Comparison of 3D DSC and 3D HD95 metrics across different bounding box expansion values using different dataset sizes for SAM fine-tuning with the image encoder frozen.

In the LoRA-based fine-tuning strategy, LoRA modules were inserted into the query and value projection layers of the SAM-ViT-B image encoder. The original pretrained parameters of the image encoder were frozen, while the LoRA parameters, prompt encoder, and mask decoder were trainable. The LoRA update was formulated as:

$$y=W_{0}x+\frac{\alpha}{r}BA\left( \mathrm{Dropout}\left( x \right) \right)$$

where *y* denotes the output feature, *W*_0_ denotes the frozen pretrained weight, *x* denotes the input feature to the linear projection, and *A* and *B* are trainable low-rank matrices. The LoRA rank was set to *r* = 4, the scaling factor was set to *α* = 16, and the dropout rate was set to 0.1.

**Table S1** Evaluation results (mean ± SD) of LoRA-based fine-tuning of SAM under varying box expansion margins (0–1.5 cm).

| Prompt expansion (cm) | 2D DSC | 3D DSC | 2D HD95  (mm) | 3D HD95  (mm) |
| --- | --- | --- | --- | --- |
| 0 | 0.92±0.02 | 0.92±0.02 | 3.28±1.19 | 2.75±1.33 |
| 0.1 | 0.92±0.02 | 0.93±0.02 | 3.07±1.19 | 2.57±1.33 |
| 0.2 | 0.93±0.02 | 0.93±0.02 | 2.91±1.16 | 2.46±1.35 |
| 0.3 | 0.93±0.02 | 0.93±0.02 | 2.80±1.17 | 2.36±1.39 |
| 0.4 | 0.93±0.02 | 0.94±0.02 | 2.80±1.38 | 2.30±1.40 |
| 0.5 | 0.93±0.02 | 0.94±0.02 | 2.86±1.50 | 2.33±1.45 |
| 0.6 | 0.93±0.02 | 0.94±0.02 | 3.01±1.67 | 2.36±1.50 |
| 0.7 | 0.93±0.02 | 0.93±0.02 | 3.18±1.74 | 2.43±1.57 |
| 0.8 | 0.93±0.02 | 0.93±0.02 | 3.32±1.84 | 2.55±1.62 |
| 0.9 | 0.93±0.02 | 0.93±0.02 | 3.47±1.96 | 2.63±1.69 |
| 1.0 | 0.92±0.03 | 0.93±0.03 | 3.70±2.13 | 2.79±1.82 |
| 1.2 | 0.92±0.03 | 0.92±0.03 | 4.28±2.56 | 3.43±2.52 |
| 1.5 | 0.91±0.04 | 0.92±0.04 | 5.53±3.72 | 4.89±3.58 |

** Note: LoRA-based fine-tuning of SAM refers to a fine-tuning strategy in which LoRA modules were inserted into the image encoder. During training, within the image encoder, only the LoRA modules were trainable, while the prompt encoder and mask decoder remained trainable.*

**Figure S2** Comparison of 3D DSC and 3D HD95 metrics across different bounding box expansion values for five fine-tuning strategies of the SAM-ViT-B model. TrainAll: training all parameters; Freeze_image_encoder: the image encoder was frozen; Freeze_mask_decoder: the mask decoder was frozen; Freeze_encoder_decoder: both the image encoder and mask decoder were frozen. LoRA: LoRA modules were inserted into the image encoder; the trainable components included the LoRA parameters, prompt encoder, and mask decoder.
